# Клиническо-лабораторные особенности наследственных феохромоцитом и параганглиом

**DOI:** 10.14341/probl12834

**Published:** 2021-11-15

**Authors:** Д. В. Реброва, Н. В. Ворохобина, Е. Н. Имянитов, В. Ф. Русаков, Л. М. Краснов, И. В. Слепцов, Р. А. Черников, Е. А. Федоров, А. А. Семенов, И. К. Чинчук, И.. В. Саблин, М. А. Алексеев, О. В. Кулешов, Ю. Н. Федотов

**Affiliations:** Санкт-Петербургский государственный университет, Клиника высоких медицинских технологий им. Н.И. Пирогова; Северо-Западный государственный медицинский университет им. И.И. Мечникова; Национальный медицинский исследовательский центр онкологии им. Н.Н. Петрова; Санкт-Петербургский государственный университет, Клиника высоких медицинских технологий им. Н.И. Пирогова; Санкт-Петербургский государственный университет, Клиника высоких медицинских технологий им. Н.И. Пирогова; Санкт-Петербургский государственный университет, Клиника высоких медицинских технологий им. Н.И. Пирогова; Санкт-Петербургский государственный университет, Клиника высоких медицинских технологий им. Н.И. Пирогова; Санкт-Петербургский государственный университет, Клиника высоких медицинских технологий им. Н.И. Пирогова; Санкт-Петербургский государственный университет, Клиника высоких медицинских технологий им. Н.И. Пирогова; Санкт-Петербургский государственный университет, Клиника высоких медицинских технологий им. Н.И. Пирогова; Санкт-Петербургский государственный университет, Клиника высоких медицинских технологий им. Н.И. Пирогова; Санкт-Петербургский государственный университет, Клиника высоких медицинских технологий им. Н.И. Пирогова; Санкт-Петербургский государственный университет, Клиника высоких медицинских технологий им. Н.И. Пирогова; Санкт-Петербургский государственный университет, Клиника высоких медицинских технологий им. Н.И. Пирогова

**Keywords:** феохромоцитома, параганглиома, синдром множественной эндокринной неоплазии 2 и 3 типов, нейрофиброматоз 1 типа, синдром фон Гиппеля — Линдау, синдром наследственных параганглиом.

## Abstract

Широкое внедрение в клиническую практику молекулярно-генетических исследований позволило определить, что более трети всех случаев катехоламин-продуцирующих хромаффинных опухолей обусловлены герминальными (наследственными) мутациями. Несмотря на многообразие клинических проявлений феохромоцитом и параганглиом (ФХЦ/ПГ), существует достаточное количество клинико-лабораторных ориентиров, позволяющих предположить наследственный генез заболевания и даже конкретный синдром. В их числе отягощенный семейный анамнез, возраст пациента, наличие сопутствующих состояний, симптомы заболевания. Необходимо учитывать, что каждая из мутаций у пациентов с ФХЦ/ПГ ассоциирована с развитием определенных заболеваний, что определяет тактику лечения и обследования пациента. Например, у таких пациентов имеется высокий риск развития злокачественных новообразований различной локализации. Информированность практического врача об особенностях течения семейных форм ФХЦ/ПГ позволит совершенствовать тактику ведения этих больных.

В статье приведены актуальные сведения о распространенности наследственных форм ФХЦ/ПГ, освещены современные взгляды на патогенез заболевания при различных мутациях, описаны основные наследственные синдромы, ассоциированные с ФХЦ/ПГ: синдромы множественной эндокринной неоплазии 2 и 3 типов, нейрофиброматоз 1 типа, синдром фон Гиппеля — Линдау, синдром наследственных параганглиом, а также клинико-лабораторные особенности при этих заболеваниях. В обзоре рассмотрены основные позиции по вопросу необходимости проведения генетического скрининга у пациентов с ФХЦ/ПГ.

## Abstract (en)

The widespread introduction of genetic testing in recent years has made it possible to determine that more than a third of cases of pheochromocytomas and paragangliomas (PPPGs) are caused by germline mutations. Despite the variety of catecholamine-producing tumors manifestations, there is a sufficient number of clinical and laboratory landmarks that suggest a hereditary genesis of the disease and even a specific syndrome. These include a family history, age of patient, presence of concomitant conditions, and symptoms of the disease. Considering that each of the mutations is associated with certain diseases that often determine tactics of treatment and examination of a patient, e.g. high risk of various malignancies. Awareness of the practitioner on the peculiarities of the course of family forms of PPPGs will allow improving the tactics of managing these patients.

The article provides up-to-date information on the prevalence of hereditary PPPGs. The modern views on the pathogenesis of the disease induced by different mutations are presented. The main hereditary syndromes associated with PPPGs are described, including multiple endocrine neoplasia syndrome type 2A and 2B, type 1 neurofibromatosis, von Hippel-Lindau syndrome, hereditary paraganglioma syndrome, as well as clinical and laboratory features of the tumor in these conditions. The main positions on the necessity of genetic screening in patients with PPPGs are given.

Феохромоцитома (ФХЦ) — это опухоль мозгового слоя надпочечника, состоящая из хромаффинных клеток, продуцирующая катехоламины (адреналин, норадреналин и дофамин), является частным случаем симпатической параганглиомы (ПГ). ПГ — это опухоль, состоящая из вненадпочечниковой хромаффинной ткани симпатических паравертебральных ганглиев грудной клетки, брюшной полости и таза. ПГ из парасимпатических ганглиев основания черепа и шеи, развивающиеся вдоль языкоглоточного и блуждающего нервов, являются в абсолютном большинстве случаев гормонально-неактивными [1].

Феохромоцитома (надпочечниковая параганглиома) и параганглиома (ФХЦ/ПГ) относятся к редким гормонально-активным нейроэндокринным опухолям. Заболеваемость составляет 2–8 случаев на 1 млн населения в год. Средний возраст пациентов — 40–50 лет [2]. В типичных случаях у больных наблюдается выраженная симптоматика заболевания, поэтому с теоретической точки зрения клиническая диагностика опухоли не должна вызывать значительных затруднений. Однако в реальной клинической практике больные с ФХЦ/ПГ могут длительное время наблюдаться у врачей различных специальностей без установленного верного диагноза и без соответствующей патогенетической терапии (по данным Ilias и Thomopoulos, в среднем до 3 лет) [2]. Поскольку в типичном случае подтверждение ФХЦ/ПГ лабораторными и современными визуализирующими методами в настоящее время достаточно доступно для любого звена здравоохранения, особое значение приобретают сведения о клинических проявлениях заболевания и информированность о них у врачей.

Одна из причин поздней диагностики ФХЦ/ПГ — значительная вариабельность симптомов как по степени выраженности (от бессимптомного течения до дебюта заболевания в форме жизнеугрожающих состояний), так и по виду. Классическими проявлениями гиперсекреции катехоламинов считаются приступы высокой артериальной гипертензии с сильнейшими головными болями, выраженная профузная потливость, сердцебиение —так называемый симпатоадреналовый криз [3][4]. Часто наблюдаются бледность кожи, тревога, боль в груди, тошнота, рвота, слабость, потеря веса, запоры и другие симптомы. Примечательно, что все эти симптомы или их сочетания являются неспецифическими. По мнению R. Stolk и соавт. и A. Geroula и соавт., отсутствие характерной клинической картины нередко приводит к тому, что измерение артериального давления в кабинете врача и факт регистрации артериальной гипертензии даже с очень высокими цифрами никак не помогают установить диагноз ФХЦ/ПГ [3][4]. Известное сочетание симптомов (артериальная гипертензия, потливость и сердцебиение), согласно современным представлениям, имеется менее чем у 25% больных с ФХЦ/ПГ [4]. Считается, что распространенность бессимптомных форм составляет около 10–17%, однако она может быть существенно недооценена, учитывая, что значительное число ФХЦ не диагностируется при жизни [5]. Учитывая, что характерная триада регистрируется примерно у 10–11% пациентов, не имеющих ФХЦ/ПГ, оценка этого параметра вряд ли будет служить сколь-нибудь надежным диагностическим признаком заболевания [3]. В связи с этим современные руководства по ФХЦ/ПГ не ориентированы на скринирование пациентов с артериальной гипертензией в отсутствие убедительных признаков опухоли [6].

Клиническую картину ФХЦ/ПГ определяют несколько вариабельных факторов: тип и характер гиперсекреции катехоламинов, чувствительность и количество адренорецепторов, сопутствующая секреция пептидных гормонов (например, кортикотропина, кальцитонина, серотонина, соматостатина, опиоидных пептидов, вазоактивного интестинального пептида, интерлейкина 6) [7][8] опухолью и размер новообразования. Сопутствующие заболевания (например, сердечно-сосудистые) также вносят вклад в изменчивость симптоматики ФХЦ/ПГ. Именно поэтому катехоламин-продуцирующие опухоли в литературе получали такие названия, как «великий имитатор» и «клинический хамелеон» [3].

Существует несколько общих закономерностей симптоматики в зависимости от гормональной активности ФХЦ/ПГ. ФХЦ чаще имеют симптоматическое течение. Они в большинстве случаев секретируют адреналин и норадреналин в различных соотношениях. Для опухолей, секретирующих преимущественно адреналин, характерны более выраженные симптомы, в особенности пароксизмальная артериальная гипертензия, тремор, бледность, тревога, сердцебиение, тахиаритмии, обмороки, гипергликемия. При новообразованиях с гиперсекрецией норадреналина чаще наблюдаются постоянное повышение артериального давления, головная боль, потливость [3, 9]. В очень редких случаях ФХЦ секретирует преимущественно или исключительно дофамин, у этих пациентов реже выявляются гипертензии и сердцебиения [10], могут возникать нетипичные для заболевания симптомы, такие как тошнота, потеря веса [11] или диарея [12]. Во многих случаях опухоль не проявляет себя клинически [5].

ПГ происходят либо из хромаффинной ткани симпатических нервных ганглиев, либо из парасимпатических нервных ганглиев. Большинство симпатических ПГ характеризуются симптоматическим течением. Абсолютное большинство симпатических ПГ протекает с гиперсекрецией норадреналина. Избыток адреналина для данного вида опухолей не характерен вследствие отсутствия фермента фенилэтаноламин-N-метилтрансферазы (PNMT). Парасимпатические ПГ чаще имеют асимптоматическое течение (около 95% случаев [13]). При отсутствии гормональной активности на первый план выходит клиническая картина, связанная с наличием объемного новообразования, сдавливающего окружающие ткани: парасимпатические ПГ локализуются в области шеи и основания черепа, иногда в верхнем средостении, симпатические — в нижнем средостении, брюшной полости, области тазовых симпатических сплетений.

Целью настоящей статьи является освещение вопросов клиническо-лабораторных особенностей ФХЦ/ПГ у пациентов с семейными формами заболевания. В последние годы отношение к этой группе пациентов было существенно пересмотрено, так как успехи, связанные с широким внедрением генетического тестирования, позволили определить около 20 генов, мутации в которых связаны с развитием ФХЦ/ПГ [9][14]. В настоящее время считается, что до 35–40% случаев развития этих опухолей обусловлено наследуемыми генетическими мутациями [15]. Во многих случаях только на основании анализа симптомов заболевания можно заподозрить наличие мутации, которая привела к развитию опухоли. Это особенно актуально, учитывая, что генетическое тестирование целой панели генов не всегда возможно реализовать на практике.

Согласно современным представлениям, с развитием ФХЦ/ПГ ассоциированы следующие генетические синдромы [14][16]:

Все указанные синдромы и соответствующие мутации могут быть разделены на 3 основные группы: псевдогипоксии, киназного сигналинга и сигналинга Wnt [16].

Патогенез заболевания при мутациях группы псевдогипоксии (SDHA, SDHB, SDHC, SDHD, SDHAF2, FH, VHL, EPAS1) связан с избыточной активацией факторов, индуцируемых гипоксией (hypoxia inducible factors, HIFs), в отсутствие гипоксии. Почти все мутации этой группы ассоциированы с гиперсекрецией ФХЦ/ПГ норадреналина и иногда дофамина, но не адреналина [10]. В целом для опухолей характерно более агрессивное течение, в частности, например, мутация SDHB в большинстве случаев приводит к инвазии и метастазированию [9][17].

Группа мутаций киназного сигналинга (RET, NF1, TMEM127, HRAS, MAX) приводит к изменению активности различных киназных сигнальных путей, таких как RAS/RAFG/MAPK и PI3K/AKT/mTOR, что способствует возникновению неоплазии. Для этих новообразований в целом характерна гиперсекреция преимущественно адреналина ФХЦ/ПГ вследствие большого количества фермента PNMT, преобразующего норадреналин в адреналин в опухоли. Норадреналин может также быть несколько повышенным. Большинство опухолей этой группы характеризуется доброкачественным течением, но при этом высокой частотой рецидивирования и мультифокальностью [18][19].

Гены CSDE1 и MAML3 приводят к онкогенезу путем активации сигнальных путей Wnt и Hedgehog. Опухоли часто продуцируют большое количество хромогранина А. Считается, что новообразования этой группы характеризуются наиболее агрессивным течением, активным метастазированием, частыми рецидивами. Тип гормональной активности при этих образованиях не установлен [19][20].

Частота и основные клинические проявления наиболее распространенных генетических синдромов, ассоциированных с ФХЦ/ПГ, приведены в таблице 1 [21].

**Table:** Таблица 1. Частота и основные клинические проявления наиболее распространенных генетических синдромов, ассоциированных с феохромоцитомой/параганглиомой.(Адаптировано из WHO classification of tumors of endocrine organs / edited by Lloyd RV, Osamura RY, Kloppel G, Rosai J. 4th edition)

Мутация гена (локус)	Предполагаемая частота	ФХЦ	ПГ груди и брюшной полости	ПГ головы и шеи	Риск метастазирования	Другие возможные клинические проявления синдрома
VHL (3p25.5)	9%	+++	Редко	Очень редко	5%	Почечно-клеточный рак, гемангиобластома, НЭО ПЖ и других органов; серозная цистаденома ПЖ, кисты почек, печени, придатка яичка, опухоли эндолимфатического мешка
RET (10q11.2)	5%	+++	Редко	Очень редко	<5%	Медуллярная карцинома ЩЖ, первичный гиперпаратиреоз, кожный лихеноидный амилоидоз, марфаноидный фенотип, невриномы слизистой оболочки, ганглионевриномы ЖКТ
NF1 (17q11.2)	2%	++	Редко	Очень редко	12%	Нейрофибромы, пятна цвета «кофе с молоком», узелки Лиша, глиома зрительного нерва, НЭО двенадцатиперстной кишки (соматостатинома), гастроинтестинальная стромальная опухоль
SDHD (11q23)	5–7%	+	++	++	<5%	Почечно-клеточный рак, гастроинтестинальная стромальная опухоль, аденома гипофиза
SDHC (1q23.3)	1–2%	Редко	Редко	++	Низкий	Почечно-клеточный рак, гастроинтестинальная стромальная опухоль
SDHB (1p36.13)	6–8%	+	+++	+	30–70%	Почечно-клеточный рак, гастроинтестинальная стромальная опухоль, аденома гипофиза
SDHA (5p15.33)	1–2%	Редко	+	+	Низкий	Почечно-клеточный рак, гастроинтестинальная стромальная опухоль, аденома гипофиза
SDHAF2 (11q12.2)	<1%	−	−	++	Низкий	Не описаны
TMEM127 (2q11.2)	1%	++	+	+	Низкий	Почечно-клеточный рак
MAX (14q23.3)	1%	+	+	+	10%	Не описаны
FH (1q42.1)	1%	+	+	+	>50%	Почечно-клеточный рак, лейомиоматоз

Характеристика опухолей, связанных с малоизученными генами, такими как TMEM127, MAX, MDH2 и другими [22], нуждается в уточнении.

## СИНДРОМЫ МНОЖЕСТВЕННОЙ ЭНДОКРИННОЙ НЕОПЛАЗИИ (МЭН) 2A И 2B ТИПОВ

Синдромы МЭН представляют собой гетерогенную группу расстройств, определяющих предрасположенность пациента к опухолям 2 или более эндокринных органов. В настоящее время выделяют 4 типа синдрома, которые наследуются аутосомно-доминантным путем. Диагноз клинически можно заподозрить либо при возникновении двух или более опухолей, ассоциированных с МЭН, либо при обнаружении одной такой опухоли при наличии родственника первой степени родства, у которого синдром установлен клинически или подтверждена ассоциированная генетическая мутация (даже при отсутствии каких-либо признаков заболевания).

Развитие ФХЦ/ПГ типично для МЭН 2A и 2B типов, при этом ПГ встречаются редко, до 1% случаев [9]. Синдром МЭН 2А типа (также известный как синдром Сиппла) включает в себя медуллярный рак щитовидной железы, ФХЦ и первичный гиперпаратиреоз, 2B типа (также известный как синдром Горлина) — медуллярный рак щитовидной железы (агрессивный и дебютирующий в молодом возрасте), ФХЦ, деформации скелета и ганглионейроматоз [23].

Распространенность синдрома МЭН 2A типа составляет примерно 1:25 000 населения. У 95% пациентов первым проявлением заболевания бывает медуллярный рак щитовидной железы [23]. Опухоль может развиваться в молодом возрасте, в связи с чем рекомендуется профилактическая тиреоидэктомия у детей с носительством ряда мутаций в экзонах 10, 11, 13–16 гена RET [23][24]. Наряду с медуллярным раком щитовидной железы в 20–30% случаях выявляется гиперпаратиреоз, протекающий обычно бессимптомно или с минимальным количеством симптомов. У 50% пациентов выявляется ФХЦ. Развитие синдрома МЭН 2A типа определяют несколько разных мутаций в гене RET [23], наиболее распространенная лоцируется в 634 кодоне [24].

Распространенность МЭН 2B типа значительно ниже в сравнении с заболеванием 2 типа, она составляет примерно 0,2:100 000 населения. В типичных случаях синдром дебютирует агрессивным медуллярным раком щитовидной железы на первой или второй декаде жизни. ФХЦ развивается примерно у 50% пациентов [23], а в детском возрасте — у 20% [25]. Часто при МЭН 2B типа выявляются другие симптомы при рождении или в раннем детском возрасте, которые могут помочь в постановке диагноза: невромы слизистых языка, губ, век, деформации опорно-двигательного аппарата, такие как марфаноподобная внешность, кифосколиоз, гипермобильность суставов, а также ганглионевроматоз органов желудочно-кишечного тракта [23]. Наиболее распространена мутация гена RET при синдроме МЭН 2B типа в 918 кодоне [24].

Для синдромов МЭН 2A и 2B типов характерны определенные клинические особенности ФХЦ. Манифестация клинических проявлений опухоли надпочечников происходит в достаточно молодом возрасте (25–32 года), в редких случаях — в возрасте от 8 до 12 лет. Медуллярный рак щитовидной железы развивается позже. Около 65% опухолей, связанных с МЭН, обнаруживаются сразу в обоих надпочечниках. ФХЦ с местной инвазией или метастазированием при МЭН 2A и 2B типов встречается редко, примерно у 3% пациентов [26]. ФХЦ обычно секретируют большое количество адреналина и имеют соответствующую клиническую картину [27].

У пациентов с установленным МЭН 2A или 2B типа без ФХЦ скрининговые мероприятия включают исследование метанефрина и норметанефрина в плазме крови или в суточной моче ежегодно, начиная с детского возраста: с 11 лет — при носительстве мутаций гена RET в кодонах 918, 634 и 883, с 16 лет — при выявлении мутации в других кодонах [28]. При выявлении повышения уровня катехоламинов рекомендуется выполнение компьютерной или магнитно-резонансной томографии. При выявлении ФХЦ необходимо оперативное лечение. Если опухоль локализуется только в одном надпочечнике, то профилактическое удаление второго надпочечника не рекомендуется, так как обычно характерен низкий риск инвазии и метастазирования [29].

## НЕЙРОФИБРОМАТОЗ 1 ТИПА

Распространенность нейрофиброматоза 1 типа, известного как болезнь фон Реклингхаузена, составляет 1:2500–1:3000 населения. Несмотря на то что тип наследования заболевания аутосомно-доминантный, а пенетрантность гена полная, только у 50% пациентов обнаруживается семейный анамнез нейрофиброматоза. Причиной этого служит то, что мутация гена NF1, ответственная за развитие заболевания, часто спорадическая [30].

При нейрофиброматозе 1 типа поражаются центральная и периферическая части нервной системы, кожа, сердечно-сосудистая система, желудочно-кишечный тракт. Заболевание в большинстве случаев можно установить на основании клинических признаков до проведения генетического исследования. У пациентов выявляется два или более признаков из нижеперечисленных [31]:

Распространенность ФХЦ при этом заболевании составляет до 5,7–7,7% всех случаев (до 20–50% при наличии артериальной гипертензии). Поражение надпочечников может носить односторонний, реже билатеральный характер [32, 33]. Возраст, при котором диагностируется ФХЦ, составляет в среднем около 53–55 лет [33]. ПГ при синдроме нейрофиброматоза встречаются очень редко. По некоторым данным, примерно у 12% пациентов (3 из 25 случаев) с ФХЦ при нейрофиброматозе 1 типа опухоль оказывается злокачественной, в других сериях наблюдений не находится подтверждения этому риску (из 12 случаев ни одна опухоль не оказалась метастатической) [33]. Для опухоли характерна гиперсекреция как адреналина, так и норадреналина [34, 35]. По данным L. Képénékian и соавт. (2016), более 80% ФХЦ при нейрофиброматозе 1 типа бессимптомные, но нахождение рядом авторов высокого риска злокачественного течения может указывать на необходимость скрининга пациентов посредством определения уровней в плазме крови или суточной моче метанефрина и норметанефрина, визуализирующего исследования надпочечников [33, 36].

## СИНДРОМ ФОН ГИППЕЛЯ–ЛИНДАУ

Синдром фон Гиппеля–Линдау представляет собой состояние, характеризующееся высоким риском развития опухолей центральной нервной системы и висцеральных органов. Синдром развивается вследствие инактивирующих мутаций в гене VHL на участке хромосомы 3р25.5, наследуемых аутосомно-доминантным путем, с пенетрантностью более 90% [37]. Интересно, что примерно у 20% пациентов синдром фон Гиппеля–Линдау развивается в результате вновь возникшей мутации, без отягощенного семейного анамнеза [38]. Распространенность синдрома составляет около 1:36 000 населения [37].

Первые клинические признаки синдрома фон Гиппеля–Линдау появляются в среднем в возрасте 25–26 лет в виде доброкачественных или злокачественных опухолей или кист различных органов. Наиболее часто встречаются гемангиобластомы сетчатки, головного и спинного мозга, почечно-клеточный рак, кисты почек, ФХЦ. Описаны случаи симпатических ПГ, нейроэндокринных опухолей поджелудочной железы, новообразований внутреннего уха, кисты и цистаденомы эпидидимиса и широкой связки.

Выделяют несколько вариантов течения заболевания: при 1 типе не встречается ФХЦ, при 2А типе характерны ФХЦ и опухоли центральной нервной системы, при 2B типе — то же, что при 2А типе, а также почечно-клеточный рак, при 2С типе — только ФХЦ. При вторых типах заболевания опухоль надпочечника встречается примерно у 20–30% пациентов [35, 39]. ФХЦ при синдроме фон Гиппеля–Линдау обычно определяется в молодом возрасте. Важно, что ФХЦ/ПГ в детском возрасте примерно в 50% случаев обусловлены синдромом фон Гиппеля–Линдау [40]. Клиническими особенностями опухоли при мутации VHL являются билатеральная локализация, редкое метастазирование (до 5% случаев). В типичных случаях ФХЦ преимущественно секретируют норадреналин. Артериальная гипертензия и другие симптомы, характерные для состояний с гиперпродукцией катехоламинов, не выражены [14, 15, 39].

## НАСЛЕДСТВЕННЫЙ СИНДРОМ ПАРАГАНГЛИОМ

Основным проявлением данного синдрома с аутосомно-доминантным типом наследования является наличие ПГ. Выделяют несколько типов заболевания в зависимости от того, какая субъединица сукцинатдегидрогеназы (SDH) была инактивирована мутацией.

При 1 типе (мутация SDHD) наиболее часто (до 80% случаев) наблюдаются ПГ зоны головы и шеи. Около 74% пациентов имеют сразу несколько опухолей, как правило, доброкачественных [41, 42]. Этот вид патологии наследуется почти во всех случаях по отцовской линии (гены, наследуемые от матери, подвергаются импринтингу и не экспрессируются у потомка, пенетрантность генов отца менее 100%). Риск формирования злокачественной опухоли невелик (до 5%), характерен молодой возраст пациентов при дебюте заболевания [43].

Наследственный синдром параганглиом 2 типа обусловлен мутациями SDHAF2, которые, как и при дефектах SDHD, передаются от отцов и ассоциированы с ПГ головы и шеи. Средний возраст пациентов — 30 лет [44].

Мутация SDHC, обуславливающая синдром 3 типа, редка, ассоциирована с появлением доброкачественных ПГ головы и шеи, а также симпатических ПГ и ФХЦ. Опухоли могут быть множественными [45].

При 4 типе (мутация SDHB) возникают крупные ПГ брюшной полости и средостения и ФХЦ. Характерны инвазия и метастазирование опухолей (у 70% пациентов), часто наблюдается гиперсекреция дофамина, норадреналина. При этом типе заболевания характерно развитие почечно-клеточного рака, гастроинтестинальных стромальных опухолей, рака молочной железы, папиллярного рака щитовидной железы [16, 41]. Средний возраст пациентов составляет 30 лет, семейный анамнез заболевания часто отсутствует [46].

Гомозиготные мутации SDHA (синдром 5 типа) ассоциированы с синдромом Лея, тяжелым нейродегенеративным заболеванием, дебютирующим в детстве, а также с кардиомиопатией. Относительно недавно отмечена связь гетерозиготных мутаций с симпатическими и парасимпатическими ПГ [47]. Описано несколько случаев. Поскольку генетическая диагностика этой формы заболевания затруднена из-за наличия двух псевдогенов на хромосомах 3 и 5, истинная ее распространенность неизвестна [15]. В большинстве зарегистрированных случаев ФХЦ/ПГ, ассоциированных с мутацией SDHA, не отмечалось семейного анамнеза заболевания, опухоль не отличалась агрессивным течением [48].

Наиболее часто встречаются 1 и 4 типы заболевания.

## СЕМЕЙНЫЕ ФЕОХРОМОЦИТОМЫ/ПАРАГАНГЛИОМЫ, СВЯЗАННЫЕ С TMEM127

TMEM127 является геном — опухолевым супрессором, связанным с mTOR. Мутации ассоциированы с развитием ФХЦ, часто билатеральных. Описано большое количество мутаций гена, все из них герминальные, однако менее 20% пациентов имеют семейный анамнез заболевания. Это указывает на низкую пенетрантность мутации [49]. Возраст пациентов составляет в среднем 45 лет, риск инвазии и метастазирования опухоли мал [50].

## СЕМЕЙНЫЕ ФЕОХРОМОЦИТОМЫ И ПАРАГАНГЛИОМЫ, СВЯЗАННЫЕ С MAX

Ген MAX связан с онкогенным транскрипционным фактором MYC. Его мутации ассоциированы как с ФХЦ, которые часто оказываются двусторонними и характеризуются агрессивным ростом, так и с ПГ [51]. Патологический ген передается от отца [52], пенетрантность мутации низкая. Дебют заболевания обычно бывает после 30 лет [22].

## СИНДРОМ ПАКАКА–ЧЖУАНА

Активирующие мутации гена EPAS1 (HIF2A) вызывают синдром Пакака–Чжуана, ассоциированного с врожденной или развивающейся в детстве полицитемией, а также ФХЦ/ПГ, часто множественными, соматостатиномами [14]. Инактивирующие мутации гена EGLN1 (PHD2), гидроксилирующего HIF, по-видимому, приводят к таким же последствиям, как активация EPAS1 [22].

## СИНДРОМ НАСЛЕДСТВЕННОГО ЛЕЙОМИОМАТОЗА И ПОЧЕЧНО-КЛЕТОЧНОГО РАКА

Синдром наследственного лейомиоматоза и почечно-клеточного рака обусловлен инактивирующими мутациями гена фумаратгидратазы (FH) и является редким аутосомно-доминантным заболеванием. Встречаемость составляет примерно 1:200 000 населения [53]. Синдром наследственного лейомиоматоза и почечно-клеточного рака характеризуется частым развитием лейомиом кожи (около 70%) и матки (около 80%), а также папиллярного почечно-клеточного рака (около 35% случаев). В редких случаях (около 1%) в рамках синдрома могут встречаться ФХЦ и ПГ грудной клетки и забрюшинного пространства, более чем в 50% случаев протекающие агрессивно с метастазированием [21].

## СКРИНИНГ НАСЛЕДУЕМЫХ МУТАЦИЙ У ПАЦИЕНТОВ С ФХЦ/ПГ

Существует несколько причин проводить генетическое исследование у пациентов с ФХЦ/ПГ. Во-первых, частота опухолей, обусловленных наследуемыми мутациями, высока и достигает 40% всех случаев данных образований. Во-вторых, при многих синдромах опухоли злокачественные, с агрессивным течением. В-третьих, выявление наследственного заболевания будет способствовать раннему выявлению катехоламин-продуцирующих опухолей и других компонентов синдрома у родственников пациента. Также выявление мутаций имеет важное значение для прогнозирования течения заболевания, определения тактики ведения пациента в послеоперационном периоде.

В целом необходимо рекомендовать тестирование панели генов для каждого пациента с ФХЦ/ПГ [48]. Примерно у 13% лиц с ФХЦ/ПГ, которые удовлетворяли по крайней мере 3 из 4 критериев спорадической опухоли (отсутствие семейного анамнеза заболевания, проявлений наследственных синдромов, множественных опухолей, признаков агрессивного течения), были выявлены мутации, приведшие к развитию опухоли [54]. В то же время, учитывая стоимость секвенирования ДНК, отсутствие во многих случаях доступного центра для проведения анализа, возникает вопрос о целесообразности проведения исследования у каждого такого пациента. Нельзя не отметить, что пенетрантность многих наследственных синдромов низка. В этих случаях проведение анализа может привести к напрасным затратам службы здравоохранения и к лишней тревоге для пациента [54]. В связи с этим некоторые авторы рекомендуют проводить скрининг у определенных категорий пациентов, например, у лиц до 20 лет с ФХЦ при наличии семейного анамнеза или каких-либо признаков наследственного заболевания и у пациентов с симпатическими ПГ [55].

Наиболее важные особенности наследственных заболеваний, ассоциированных с ФХЦ/ПГ, приведенные в представленном обзоре литературы, обобщены на рисунке 1.

**Figure:**
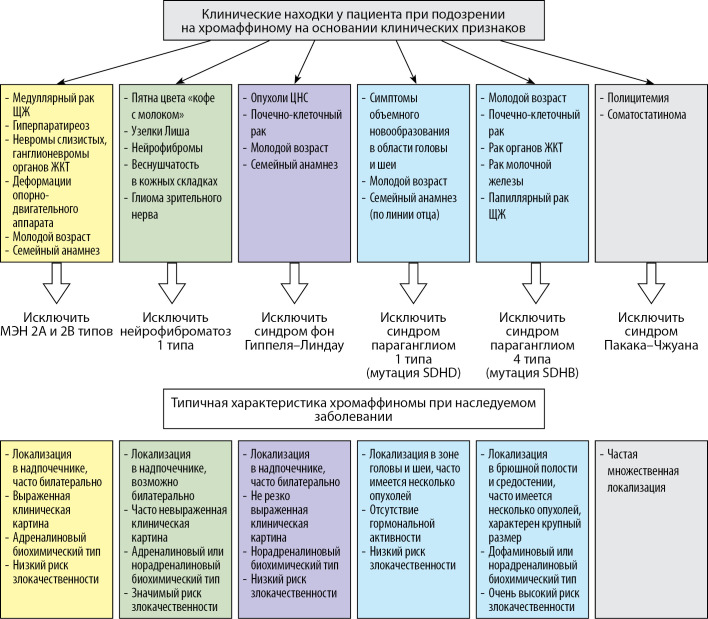
Рисунок 1. Основные клинико-лабораторные признаки генетических синдромов, ассоциированных с феохромоцитомой/параганглиомой, и типичная характеристика опухолей.Примечание: ЩЖ — щитовидная железа; ЖКТ — желудочно-кишечный тракт; ЦНС — центральная нервная система.

## ЗАКЛЮЧЕНИЕ

Широкое внедрение генетического тестирования в мире в последние годы позволило определить, что более трети ФХЦ/ПГ обусловлено мутациями и связано с тем или иным наследуемым синдромом. Наличие у пациента определенных клинико-лабораторных особенностей опухоли, а также других проявлений генетического синдрома дает основание предположить наличие конкретного наследственного заболевания, что позволяет совершенствовать подходы к терапии и обследованию пациентов с ФХЦ/ПГ.

## ДОПОЛНИТЕЛЬНАЯ ИНФОРМАЦИЯ

Источники финансирования. Работа выполнена по инициативе авторов без привлечения финансирования.

Конфликт интересов. Авторы декларируют отсутствие явных и потенциальных конфликтов интересов, связанных с содержанием настоящей статьи.

Участие авторов. Все авторы внесли существенный вклад в поиск и оценку данных литературы, написание статьи или внесение в рукопись важных правок с целью повышения научной ценности статьи. Все авторы одобрили финальную версию статьи перед публикацией, выразили согласие нести ответственность за все аспекты работы, подразумевающую надлежащее изучение и решение вопросов, связанных с точностью или добросовестностью любой части работы.
